# Cigarette smoking and metabolic syndrome components: a cross-sectional study from Maracaibo City, Venezuela

**DOI:** 10.12688/f1000research.14571.3

**Published:** 2019-01-11

**Authors:** Valmore Bermudez, Luis Carlos Olivar, Wheeler Torres, Carla Navarro, Robys Gonzalez, Cristobal Espinoza, Alicia Morocho, Andres Mindiola, Maricarmen Chacin, Victor Arias, Roberto Añez, Juan Salazar, Manuel Riaño-Garzon, Edgar Diaz-Camargo, Maria Judith Bautista, Joselyn Rojas

**Affiliations:** 1 Grupo de Investigación Altos Estudios de Frontera, Universidad Simón Bolívar, Cúcuta, Colombia; 2 Endocrine and Metabolic Diseases Research Center, School of Medicine, University of Zulia, Maracaibo, Venezuela; 3 Latacunga Province General Hospital, Ministry of Public Health, Cotopaxi, Ecuador; 4 Geriatric Research Education and Clinical Center, United States Department of Veterans Affairs, Miami, Florida, USA; 5 Division of Pulmonary and Critical Care Medicine, Brigham and Women’s Hospital and Harvard Medical School, Boston, Massachusetts, 02115, USA

**Keywords:** smoking habit, metabolic syndrome, smokers, hypertension, cardiovascular risk.

## Abstract

**Background:** A growing body of evidence suggests that cigarette smoking can cause the onset of metabolic syndrome prior to cardiovascular diseases. Therefore, the objective of this study was to evaluate the relationship between smoking habit and metabolic syndrome components in an adult population from Maracaibo city, Venezuela.

**Methods:** The Maracaibo City Metabolic Syndrome Prevalence Study is a descriptive, cross-sectional study with random and multi-stage sampling. In this sub-study, 2212 adults from both genders were selected. On the basis of their medical background, they were classified as smokers, non-smokers and former smokers. Metabolic syndrome was defined according to Harmonizing 2009 criteria, using population-specific abdominal circumference cut-off points. The association between risk factors was evaluated using a logistic regression model.

**Results:** In the studied population, 14.8% were smokers, 15.4% were former smokers. In the multivariate analysis, the presence of metabolic syndrome (smokers: OR, 1.54; 95% CI, 1.11–2.14; *p*=0.010) and its components were related to cigarette smoking, with the exception of hyperglycemia. High blood pressure was inversely associated with current smoking status (smokers: OR, 0.70 (0.51–0.95); *p*=0.025).

**Conclusion: **Cigarette smoking represents a related factor with metabolic syndrome, being associated with low high-density lipoprotein-cholesterol, increased abdominal circumference and elevated triacylglyceride levels. Former smokers did not present a greater risk for developing this metabolic disease when compared to non-smokers. The effect of avoiding this habit should be evaluated in future studies in our population.

## Introduction

Smoking is one the main causes of morbidity and mortality in the working-age population; it is responsible for approximately 7.2 million deaths per year ^1^. This constitutes a major public health issue. Almost one-third of the world population older than 15 years of age smokes ^2^, with a global prevalence of 21.2% in developing countries ^3^. In the Americas, the prevalence in the general adult population is 17.1% ^4^; however, this varies among different countries, with Chile having the highest (38.9%) and Panama the lowest (7.4%) rates ^4^. Venezuela is a country with one of the highest prevalence (33.9%) ^5^, with a frequency of 14.8% in Maracaibo City in recent studies ^6^.

Smoking habit is a major modifiable risk factor for developing non-communicable diseases ^2^, including cardiovascular disease (CVD) and type 2 diabetes mellitus (DM2) ^7^. A growing body of evidence suggests that before the onset of these two diseases, cigarette smoking favors the appearance of metabolic syndrome (MS) ^8, 9^ high blood pressure, dyslipidemia, obesity and high blood glucose ^10– 14^. Main contributors for this association include the presence of dyslipidemia and central obesity ^15^.

The physiopathology of the relationship between cigarette smoking and MS comes from a decrease in peripheral insulin sensitivity, lipoproteins metabolism alterations and endothelial dysfunction, all present in smoking individuals ^16^. Until now, epidemiological published results are not definitive in showing the association between cigarette smoking and MS. On the other hand, it is not certain whether this association is caused by other behavioral patterns and unhealthy habits of patients with cardiometabolic diseases ^17^. Thus, the aim of this study was to evaluate the relationship between smoking habit and MS components in the adult population from Maracaibo City, Venezuela.

## Methods

### Study design and subject selection

The Maracaibo city MS prevalence study (MMSPS) was a cross-sectional, descriptive study performed in Maracaibo, Venezuela. It was designed to provide estimations about the presence of MS and associated cardiovascular risk factors in the adult population during the period between May 2007 and December 2009. The study method was reported previously ^18^. The most important aspects of the protocol are presented here. Maracaibo city was divided into parishes, which were sampled proportionally through a multistage random sampling, defining conglomerates in two phases: In the first phase, the conglomerates represented the sectors of the 18 parishes, selecting 4 areas per parish by means of simple random sampling; in the second phase, the conglomerates were represented by the neighborhood of each chosen area, to which a random number was assigned. To evaluate smoking habit in this sub-study, 18 subjects were excluded: 9 because of inconsistencies about when they started smoking; the other 9 subjects because they smoked cigar types that were different to cigarettes. Finally, a total of 2212 subjects were evaluated. The study was approved by the Bioethics Committee of the Endocrine and Metabolic Research Center – University of Zulia (approval number: BEC-006-0305). This ethical approval included all future studies that used the data from the MMSPS. All participants signed an informed consent form before being questioned and physically examined by a trained team.

### Clinical evaluation

Every subject in the study underwent a medical examination performed by trained personnel to obtain a full medical history. During the anamnesis, past medical and family history of endocrine and metabolic disorders was collected; including age, race, marriage status, education and socioeconomic status. The latter was measured using the Graffar scale modified by Mendez-Castellano and De Mendez ^19^.

The auscultatory method was performed to measure arterial pressure, using an adequate calibrated and validated sphygmomanometer. Korotkoff phases I and V were used to measure systolic and diastolic pressures, respectively. Subjects remained sitting still for 15 minutes before assessment, with both feet on the ground. A total of 3 measurements per day were taken in 15 minute intervals, for 2 days consecutively. Anthropometric measures were taken using a height rod that had been previously calibrated and placed on a flat surface. Weight was measured using a digital weighing scale (Tanita, TBF-310 GS Body Composition Analyzer; Tokyo, Japan), with the patient wearing light clothes and no shoes. The body mass index (BMI) was calculated applying the Quetelec formula (weight/height ^2^), and classified according to the WHO classification ^20^, as follows: normal weight (<25 Kg/m ^2^), overweight (25.0–29.9 Kg/m ^2^), obese (≥30.0 Kg/m ^2^). Abdominal circumference was measured using a plastic measuring tape, graded in centimeters and millimeters, in a spot equidistant to the lower ribcage and the anterior-superior iliac spine, according to the United States National Institute of Health protocol ^21^.

### Smoking habit evaluation

Subjects were asked about smoking habit presence and duration, being categorized as: a) current smoker, any subject who had smoked more than 100 cigarettes in his/her lifetime, is currently smoking, or less than 1 year had passed after he/she quit smoking; b) Former smoker: any subject who has quit smoking for more than 1 year; c) non-smoker, any subject who has never smoked or had smoked less than 100 cigarettes in his/her lifetime ^6^. Smoking intensity was assessed posteriorly, according to number of cigarettes per day. It was divided in the following tertiles: T1 <3 cigarettes/day; T2 = 3–9 cigarettes/day; and T3 ≥10 cigarettes/day.

### Physical activity and alcohol consumption evaluations

Physical activity was evaluated using the International Physical Activity Questionnaire ^22^. It takes into account four elements of evaluation: physical activity in transport, work, domestic and gardening, and leisure time. To quantify time investment on each element, subjects were classified in quintiles. The final scoring was reported using metabolic equivalents (METs)-min/week on each item; any subject with 0 METs was considered as physically inactive.

Subjects with ≥1 MET were classified in quintiles according to gender, resulting in six categories for physical activity: physical inactivity (MET = 0), very low (Q1), low (Q2), moderate (Q3), high (Q4), and very high (Q5) physical activity. Leisure time was classified as: a) Q1 or very low physical activity, <296.999 METs for men and <230.999 METs for women; b) Q2 or low physical activity, 297.000–791.999 METs for men and 231.000-445.499 METs for women; Q3 or moderate physical activity, 792.000–1532.399 METs for men and 445.500-742.499 METs for women; Q4 or high physical activity, 1532.400–2879.999 MET for men and 742,500–1798.499 METs for women; and e) Q5 or very high physical activity, ≥2879.000 METs for men and ≥1798.500 METs for women. For alcohol consumption, any subject that drinks ≥1 gram daily was considered as a “drinker” ^23^.

### Laboratory analysis

After 8 hours of fasting, a blood sample was taken from the cubital vein, and was centrifuged to obtain the serum. Serum levels of glucose (catalog number REF-10123), total cholesterol (catalog number REF-10015) and triacylglycerides (TAG) (catalog number REF-10163) were determined using enzymatic–colorimetric kits (Human Gesellschaft für Biochemica und Diagnostica mbH) and a specialized computer system. Glycemic status was classified according to ADA 2017 criteria in normal glucose (basal glucose, <100 mg/dl), impaired fasting glucose (basal glucose, 100–125 mg/dl) and DM2 (≥126 mg/dl) ^24^. Serum hs-C reactive protein (hs-CRP) levels were quantified using immunoturbodimetric assays (Human Gesellschaft für Biochemica und Diagnostica mbH. (catalog number REF-11544), setting the cutoff point at ≥0,765 mg/l ^25^.

Basal insulin serum levels were determined using a commercial kit (catalog number EIA-2935) based on the ELISA method (DRG International, Inc.), with a detection limit of <1 mU/l. Insulin resistance (IR) was calculated using software ( HOMA-Calculator v2.2.2) supplied by the Oxford Centre for Diabetes, Endocrinology and Metabolism; the cutoff-point for HOMA2-IR was 2.00 ^26^.

### Metabolic syndrome evaluation

MS diagnosis was made using the proposed criteria from the IDF and AHA/NHLBI in 2009 ^27^. It requires three or more of the following components to achieve a diagnosis: 1) TAG ≥150 mg/dl; 2) high-density lipoprotein–cholesterol (HDL-C) <40 mg/dl for men or <50 mg/dl for women; 3) basal glucose levels ≥100 mg/dl, or a previous diagnosis of DM2 or use of an antidiabetic drug; 4) arterial pressure ≥130/85 mmHg, or a previous diagnosis of hypertension or use of an antihypertensive drug; 5) abdominal circumference with cutoff points adapted for our population, which are ≥91 for women and ≥98 cm for men ^28^.

### Statistical analysis

Qualitative variables were expressed in absolute and relative frequencies. The relationship between these was examined with a χ ^2 ^test and the difference in proportions using a Z-test. Quantitative variables were expressed in arithmetic means ± standard deviations, with prior analysis using Geary’s test. Variables without a normal distribution were submitted to logarithmic transformation with posterior normality test. Multiple logistic regression models were made to estimate odds ratios (OR) and 95% confidence intervals (95% CI); they were used for the presence of MS and each of its components, adjusted for gender, age, ethnic group, marital status, education level, socioeconomic status, working status, alcohol consumption, BMI categories, insulin resistance and smoking habit. On another model, smoking intensity was assessed dividing consumption in tertiles (T1 <3 cigarettes/day; T2 = 3–9 cigarettes/day; and T3 ≥10 cigarettes/day). Data were analyzed by using SPSS v.21 for Windows (IBM SPSS), and considering statistically significant results when *p*<0,05.

## Results

### General characteristics of the sample

A total of 2212 individuals were studied, of whom 52.7% (n=1166) were women. The mean age ± SD was 39.27±15.38 years, and the most frequently occurring age group was 30–49 years (38.5%; n=851). For smoking habit, 14.8% were smokers (n=328), 15.4% former smokers (n=340) and 69.8% were non-smokers (n=1544). The prevalence of MS was 35.7% (n=935) in the sample. The most frequent MS components were low HDL-C (57.6%; n=1275) and abdominal obesity (48.5%; n=1072). Other general characteristics can be found in Table 1.

**Table 1.** General characteristics of the sample population.

Variable	Female (n=1166)		Male (n=1046)		Total (n=2212)	
	n	%	n	%	n	%
Age (years)						
<30	349	29.9	410	39.2	759	34.3
30–49	464	39.8	387	37.0	851	38.5
≥50	353	30.3	249	23.8	602	27.2
Marital status						
Single	495	42.5	455	43.5	950	42.9
Married	427	36.6	446	42.6	873	39.5
Other	244	20.9	145	13.9	389	17.6
Socioeconomic status						
Stratus I: high class	17	1.5	19	1.8	36	1.6
Stratus II: middle-high class	206	17.7	204	19.5	410	18.5
Stratus III: middle class	432	37.0	440	42.1	872	39.4
Stratus IV: Working class	446	38.3	344	32.9	790	35.7
Stratus V: extreme poverty	65	5.6	39	3.7	104	4.7
Working status						
Employed	527	45.2	757	72.4	1284	58.0
Unemployed	639	54.8	289	27.6	928	42.0
Education						
Illiterate	33	2.8	19	1.8	52	2.4
Primary school	237	20.3	112	10.7	349	15.8
High school	516	44.3	519	49.6	1035	46.8
Higher education	380	32.6	396	37.9	776	35.1
Ethnic group						
Mixed	873	74.9	807	77.2	1680	75.9
White-Hispanic	189	16.2	159	15.2	348	15.7
Afro-Venezuelan	30	2.6	36	3.4	66	3.0
Indigenous American	62	5.3	43	4.1	105	4.7
Other	12	1.0	1	0.1	13	0.6
Smoking habit						
Non smoker	878	75.3	666	63.7	1544	69.8
Current smoker	134	11.5	194	18.5	328	14.8
Former smoker	154	13.2	186	17.8	340	15.4
Leisure time physical activity						
Inactive	832	71.4	529	50.9	1361	61.7
Very low	67	5.7	99	9.5	166	7.5
Low	62	5.3	102	9.8	164	7.4
Moderate	64	5.5	103	9.9	167	7.6
High	72	6.2	92	8.8	164	7.4
Very high	69	5.9	115	11.1	184	8.3
Alcohol consumption	219	18.8	505	48.3	724	32.7
Metabolic syndrome †	470	40.3	465	44.5	935	42.3
Metabolic syndrome ‡	391	33.5	398	38.0	789	35.7
Low HDL †	747	64.1	528	50.5	1275	57.6
High TAG †	268	23.0	343	32.8	611	27.6
Hyperglycemia †	301	25.8	317	30.3	618	27.9
High blood pressure †	406	34.8	447	42.7	853	38.6
Abdominal obesity †	920	78.9	741	70.8	1661	75.1
Abdominal obesity ‡	569	48.8	503	48.1	1072	48.5

WC, waist circumference; HDL, high-density lipoprotein; TAG, triacylglycerides. †According to IDF and AHA/NHLBI-ISO-2009 Consensus ^27^. ‡ According to The Maracaibo City Metabolic Syndrome Study ^28^: WC ≥98cm for men; ≥91cm for women.

### Smoking habit and metabolic syndrome components

Smoking habit in accordance to MS components could be seen in Table 2. It shows a statistically significant association between cigarette smoking and having MS (χ ^2^=39.285; *p<*0.001) with a greater percentage of individuals with MS in former smokers (47.9%) and current smokers (42.1%) than in non-smokers (31.6%), *p*<0.05.

**Table 2.** Smoking habit in accordance with MS and its components. Maracaibo, Venezuela.

Smoking status	MS ‡		High TAG †		Abdominal obesity ‡		Hyperglycemia †		Low HDL-C †		High BP †	
	n	%	n	%	n	%	n	%	n	%	n	%
Nonsmoker	488	31.6	364	23.6	687	44.5	400	25.9	878	56.9	569	36.9
Current Smoker	138	42.1	121	36.9	171	52.1	104	31.7	201	61.3	119	36.3
Former Smoker	163	47.9	126	37.1	214	62.9	114	33.5	196	57.6	165	48.5

MS, Metabolic Syndrome; TAG, triacylglycerides; HDL-C, high-density lipoprotein-cholesterol; BP, blood pressure. MS: χ ^2^ *=* 39.285 (p<0.001); High TAG: χ ^2^ *=* 41.886 (p<0.001); Abdominal Obesity: χ ^2^ *=* 40.039 (p<0.001); Hyperglycemia: χ ^2^ *=* 10.759 (p=0.005); Low HDL-C levels: χ ^2^ *=* 2.160 (p=0.340); High Blood Pressure: χ ^2^ *=* 16.883 (p<0.001). †According to IDF and AHA-NHLBI-ISO 2009 ^27^. ‡ According to The Maracaibo City Metabolic Syndrome Study ^28^: WC ≥98cm for men; ≥91cm for women.

Each component of the MS was analyzed in relation to smoking. A higher percentage of individuals with high TAG were former (37.1%) and current (36.9%) smokers, compared with non-smokers (23.6%) (χ ^2^=41.886; *p*<0.001). The same happened for abdominal obesity in former smokers (62.9%) and current smokers (52.1%) (χ ^2^=40.039, *p*<0.001). A high percentage of former smokers presented hyperglycemia (33.5% vs 25.9%; χ ^2^=10.759; *p*<0.005) and high blood pressure (48.5 vs 36.9%; χ ^2^=16.88; *p*<0.001) in comparison to nonsmokers. No statistical association was found between low HDL-C and smoking status.

**Figure 1.**
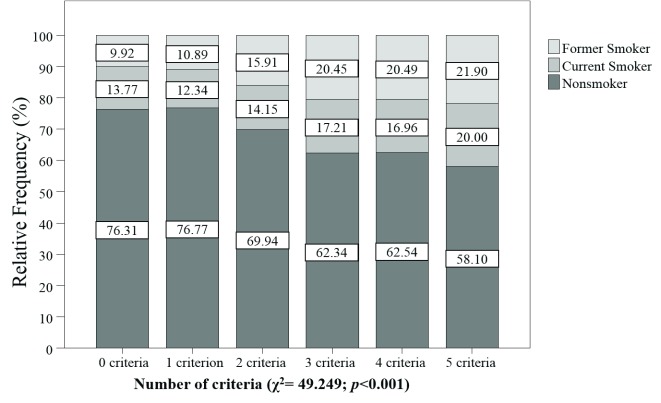
Smoking habit and number of metabolic syndrome criteria in subjects from Maracaibo, Venezuela.

Comparing smoking habit with the number of MS criteria ( Figure 1), a statistically significant association was observed (χ ^2^=49.249, *p*<0.001). The highest percentages were with nonsmokers who met 0 criteria (76.31%) and 1 criterion (76.77%). However, the greatest prevalence of smokers was observed in subjects who met 4 (16.96%) and 5 (20%) criteria.

### Smoking habit as a risk factor for MS and its components

In Table 3, models of multivariate logistic regression are shown for the diagnosis of MS and its components. An association between current smoking and increased risk of presenting with MS could be observed (OR, 1.54; 95% CI, 1.11–2.14; *p*=0.010); the same was true of high TAG serum levels (OR, 1.66; 95% CI, 1.23–2.23; *p<*0.001); abdominal obesity (OR, 1.54; 95% CI, 1.05–2.28; *p=*0.027) and low HDL-C levels (OR, 1.32; 95% CI, 1.01–1.74; *p*=0.046).

**Table 3.** Adjusted OR for metabolic syndrome and its components according to smoking habit in Maracaibo, Venezuela.

Smoking status	MS ‡	High TAG †	Abdominal Obesity ‡	Hyperglycemia †	Low HDL-C †	High BP †
	OR (95% CI); *p*	OR (95% CI); *p*	OR (95%CI); *p*	OR (95%CI); *p*	OR (95%CI); *p*	OR (95%CI); *p*
Non- smokers	1.00	1.00	1.00	1.00	1.00	1.00
Current smokers	1.54 (1.11–2.14); 0.010	1.66 (1.23–2.23); <0.001	1.54 (1.05–2.28); 0.027	1.13 (0.83–1.54); 0.408	1.32 (1.01–1.74); 0.046	0.70 (0.51–0.95); 0.025
Former smokers	0.96 (0.70–1.31); 0.799	1.15 (0.86–1.53); 0.324	1.24 (0.85–1.81); 0.245	0.86 (0.64–1.16); 0.330	0.97 (0.74–1.26); 0.832	0.79 (0.58–1.06); 0.127

TAG, triacylglycerides; HDL-C, high-density lipoprotein-cholesterol; BP, blood pressure; OR, odds ratio; CI, confidence interval. †According to IDF and AHA/NHLBI-ISO 2009 ^27^. ‡ According to The Maracaibo City Metabolic Syndrome Study ^28^: WC ≥98cm for men; ≥91cm for women.

On the other hand, by assessing smoking intensity according to number of cigarettes per day ( Table 4), an association between the consumption tertile and high serum TAG levels was observed (T3: OR, 1.51; 95% CI, 1.03–2.22; *p=*0.036). Also, an association was observed between smoking intensity and abdominal obesity (T3: OR, 2.05; 95% CI, 1.15–3.64; *p=*0.015). By contrast, an inverse relationship was observed with high blood pressure (T3: OR, 0.66; 95% CI, 0.44–0.99; *p=*0.045).

**Table 4.** Adjusted odds ratios for MS and its components according to smoking habit intensity in Maracaibo, Venezuela.

Smoking status	MS ‡	High TAG †	Abdominal Obesity ‡	Hyperglycemia †	Low HDL-C †	High BP †
	OR (95%CI); *p*	OR (95%CI); *p*	OR (95%CI); *p*	OR (95%CI); *p*	OR (95%CI); *p*	OR (95%CI); *p*
Non-smoker	1.00	1.00	1.00	1.00	1.00	1.00
<3 cigarettes/day	1.13 (0.79–1.62); 0.487	1.25 (0.90–1.73); 0.178	1.42 (0.94–2.13); 0.092	1.02 (0.72–1.42); 0.932	1.10 (0.82–1.47); 0.539	0.75 (0.54–1.06); 0.101
3–9 cigarettes/day	1.25 (0.88–1.78); 0.208	1.41 (1.02–1.95); 0.035	1.11 (0.73–1.69); 0.620	1.01 (0.72–1.41); 0.968	1.24 (0.91–1.69); 0.166	0.81 (0.58–1.13); 0.210
≥10 cigarettes/day	1.21 (0.78–1.88); 0.381	1.51 (1.03–2.22); 0.036	2.05 (1.15–3.64); 0.015	0.90 (0.61–1.35); 0.620	1.02 (0.70–1.49); 0.912	0.66 (0.44–0.99); 0.045

MS, metabolic syndrome; TAG, triacylglycerides; HDL-C, high-density lipoprotein-cholesterol; BP, blood pressure. †According to IDF and AHA/NHLBI-ISO 2009 ^27^. ‡According to The Maracaibo City Metabolic Syndrome Study ^28^: WC ≥98cm for men; ≥91cm for women.

Cigarette smoking and MS MMSPS dataset Click here for additional data file. Copyright: © 2019 Bermudez V et al. 2019 Data associated with the article are available under the terms of the Creative Commons Zero "No rights reserved" data waiver (CC0 1.0 Public domain dedication).

## Discussion

Cigarettes are composed of more than 1000 toxic and carcinogenic elements. Nicotine is the main alkaloid in tobacco; it constitutes 1.5% of the commercial cigarette weight and 95% of the total alkaloids present ^29^. Despite its effects, cigarette smoking has spread all over the globe, becoming a leading cause of chronic and degenerative pathologies. In Latin America, its use has markedly increased since 1950, and is now considered the second most common cardiovascular risk factor, following high blood pressure ^30^. This has led to an increase in cancer deaths and a drop in life expectation of 2–6 years ^31^. In Maracaibo, high prevalence of cigarette smoking and MS has been observed, which may suggest an existing relationship between these variables ^6, 32^. The main finding in this report is the relationship between cigarette smoking and metabolic syndrome, being associated specially with low high-density lipoprotein-cholesterol, increased abdominal circumference and elevated triacylglyceride levels.

In this study, MS prevalence in current smokers was 42.1% in both genders and a greater probability of having MS than in nonsmokers was observed. Kang and Song in the Korea National Health and Nutrition Examination Survey (KHANES) reported similar results with a cross-sectional study with 11559 subjects. They evaluated smoking habit by looking for nicotine in urine samples; a greater risk for developing MS was observed in those subjects ^33^. Likewise, Slagter *et al.* ^11^ conducted a study in the Netherlands which included 59,467 subjects from both sexes. In that study, a higher prevalence of MS was observed in smokers (a dose-dependent relationship), and increased the risk of MS depended neither on BMI nor gender.

Sun *et al.* ^8^ conducted a meta-analysis from multiple cohort studies and included 13 articles. In total, 56,691 subjects and 8688 cases from Asia, Europe and North America were included. They found that cigarette smoking actively increases the risk of having MS. The effects of smoking on the cardiovascular system could be caused by increased action of nicotinic receptors. Activation of nicotinic receptor could promote the release of neutransmitters and hormones such as vasopressin, CRH, ACTH, growth hormone, dopamine, serotonin, glutamate and GABA in the central nervous system, acetylcholine in the peripheral nervous system, and catecholamine and cortisol from the adrenal glands. All of these molecules affect metabolism and appetite regulation ^34^.

The CKB cohort study ^35^ included 487,527 adult subjects and reported that regular cigarette smoking was associated with a decrease in BMI and an increase in abdominal circumference in both men and women (they used an adjusted model for BMI). Similar results were reported from the FINRISK study ^36^, which included 5817 Finnish adults; greater abdominal circumference was observed in overweight and obese women who smoke. Clair *et al.* ^37^, in a cross-sectional study that included 6123 adult Caucasians from Switzerland, reported that both sexes had an increased risk of obesity according to the number of cigarettes they smoke per day. These results resemble those from the present study, where cigarette smoking was associated with increased abdominal obesity. This epidemiological behavior could be explained by the recent hypothesis of the association between cigarette smoking and a decrease in body weight, using the CHRNA3 genetic variant (rs1051730); establishing that smoking does not affect body fat distribution and the increase in localized visceral fat and in abdominal obesity are due to high cortisol plasma levels and insulin resistance, respectively ^38– 40^.

In this study, cigarette smoking represents a risk factor for having high TAG levels. Similar behavior was seen in the ICMR-INDIAB cross-sectional study ^41^ of 16,607 adult individuals, which showed a positive correlation between high TAG levels and smoking. Ueyama *et al.* ^42^ reported there was a positive association between smoking and high TAG levels in a study of 5959 Japanese individuals. This phenomenon could be explained by the fact that stimulation of the sympathetic nervous system produces the release of insulin antagonists. These antagonists, such as cortisol and growth hormone, increase lipolysis, leading to an elevation of free fatty acids in the blood ^8, 40^.

Low HDL-C levels were observed more frequent in smokers than nonsmokers in our study. Sun *et al.* ^43^, in a cross-sectional study of 11,956 Chinese individuals, reported similar results by showing that current smokers had an increased risk of having low HDL-C levels. Takata *et al.* ^44^, showed that in 32 individuals who were participating in an anti-smoking program using varenicline or transdermal nicotine patches, HDL-C levels, apolipoprotein AI and HDL subfractions did not change significantly according to therapeutic strategy used. In the same study, cholesterol efflux capacity and HDL inflammatory index improved significantly with the anti-smoking program (baseline cholesterol efflux capacity: 14.15±2.46% vs after smoking cessation cholesterol efflux capacity: 14.83±2.35%; *p*=0.01; baseline HDL inflammatory index: 1.13±0.31 vs after smoking cessation HDL inflammatory index: 0.98±0.18 %; *p*=0.01).

The inverse relationship between current cigarette smoking and high blood pressure observed in the present study is noteworthy. However, three decades ago cigarette smoking was globally reported as acutely increasing blood pressure, heart frequency and myocardial contractility ^45^. This was thought to be caused by increased nicotinic activity on the sympathetic nervous system. Despite this, epidemiological evidence could not confirm the role of cigarette smoking in the development of elevated blood pressure ^46^. On the other hand, diverse evidence suggests an inverse relationship between these factors. Kaneko *et al.* ^47^, in a recent study of 1297 Japanese individuals without any history of high blood pressure, showed that cigarette smoking appeared to be a “protective” factor against blood pressure elevation. Onat *et al.* ^48^ observed a similar pattern in a Turkish population. The inverse association between blood pressure and smoking habit could be related to the cigarette effect on weight loss, since obesity is associated with a high incidence of high blood pressure; explaining the rebound effect on blood pressure in obese subjects who stop smoking ^48^; however, this study evidenced that smokers presented with more abdominal obesity than nonsmokers. Another theory to explain this behavior suggests that smokers show less response to psychological stress: many of them report a decrease in anxiety and stress when smoking a cigarette ^49^. This may come from modifications to adrenal and cardiovascular responses to external stimuli caused by cigarette smoking; thus, stopping smoking would increase blood pressure ^50^.

Leone ^51^ reports a two-phase effect of cigarette smoking on arterial pressure: the first phase, without a determined duration, when there is a decrease in blood pressure; and the second phase, when the smoker develops elevated blood pressure from the toxic effects of carbon monoxide ^51^. This finding shows the importance in chronologically assessing smoking habit duration. Despite this, smoking does not benefit to cardiovascular health, but increases the risk of cardiovascular disease, especially in men ^48^. These are not the only contradictory findings in relation to tobacco use and the presence of hypertension, recently Gonzalez *et al*. ^52^ reported in a population from The Andes region of Venezuela, an association between the "chimó" consumption (a smokeless tobacco preparation) and lower frequency of hypertension, suggesting that the occurrence of masked hypertension in tobacco users as a possible explanation-an issue that is highly probable given the prejudices known to patients when using these products. Therefore, analyses with ABMP are necessary to assess the effect of tobacco on blood pressure throughout the day.

Similarly, with the assessment of smoking intensity according to number of cigarettes per day, a direct relationship was found between the number of cigarettes smoked and an increased risk of high serum TAG levels and abdominal obesity, and an inverse relationship with hypertension; this was seen especially in heavy smokers (≥10 cigarette daily). In this sense, in a study performed by Chen *et al.* ^10^, 1146 individuals showed a significant dose-response relationship between the number of cigarettes per day and high TAG levels. Data analysis from the KHANES study revealed an increased risk of obesity and central obesity with an increase in smoking habit intensity ^53^. This relationship could be caused by the dose-dependent effect of nicotine on fatty acid metabolism and catecholamine release; also inducing increase in lipolysis, free fatty acids, VLDL, LDL levels, and visceral adipose tissue independent of weight gain or loss ^54^.

In the present study, former smokers did not exhibit an increased risk of developing MS or its components when compared with non-smokers. Similar results were reported in Korea by Oh *et al.* ^55^. The benefits to cardiovascular health from stopping cigarette smoking seem to depend on the following variables: first, the time since the subject stopped; and second, the length of time for which he/she was smoking and the quantity of cigarettes. A previous study showed that smoking 20 cigarettes daily increased the risk of developing MS for the next 10 years, whereas smoking 40 cigarettes daily increased the risk for the next 20 years ^56^. This is why in the Maracaibo population it is necessary to conduct a cohort study on subjects who stopped smoking to evaluate the long term effects on cardiometabolic health.

Regarding the limitations of this study, its cross-sectional design makes it incapable of determining causality; it is also influenced by the subjectivity of its participants regarding the intensity and duration of their smoking habit. All of this should be considered in future studies.

In conclusion, the present study showed that smoking in our population represent a related factor with MS, and is individually associated with low HDL-C levels, increased abdominal circumference and high TAG levels. Former smokers did not show any increase in risk of present MS relative to non-smokers; despite this, future research studies should be conducted to evaluate how stopping cigarette smoking decreases cardiometabolic risk. Prevention measures focused on patients who smoke, especially anti-smoking counseling from medical personnel, could help to decrease any cigarette cardiometabolic consequences in the Maracaibo City population.

## Data availability

The data referenced by this article are under copyright with the following copyright statement: Copyright: © 2019 Bermudez V et al.

Data associated with the article are available under the terms of the Creative Commons Zero "No rights reserved" data waiver (CC0 1.0 Public domain dedication). 

**Dataset 1. Cigarette smoking and MS MMSPS dataset.** DOI: 10.5256/f1000research.14571.d201851 ^57^
