# Supplementary material for: Cigarette smoking and metabolic syndrome components: a cross-sectional study from Maracaibo City, Venezuela
Source: F1000Res. 2019 Jan 11;7:565. Originally published 2018 May 10. [Version 3] doi: 10.12688/f1000research.14571.3 (PMC6343224; doi:10.12688/f1000research.14571.3)
Supplement: Peer review history

# Peer review: Referee response for version 3

**DOI:** 10.5256/f1000research.19517.r42914

**Published:** 2019-01-21

**Authors:** Eiji Oda

**Affiliations:** 1 Medical Check-up Center, Tachikawa General Hospital, Nagaoka, Japan

I approve this revision. However, I think no new interesting findings are observed in this study.

I have read this submission. I believe that I have an appropriate level of expertise to confirm that it is of an acceptable scientific standard.

# Peer review: Referee response for version 2

**DOI:** 10.5256/f1000research.16385.r42390

**Published:** 2019-01-07

**Authors:** Juan P. González-Rivas

**Affiliations:** 1 The Andes Clinic of Cardio-Metabolic Studies, Mérida, Venezuela; 2 Foundation for Clinical, Public Health, and Epidemiological Research of Venezuela (FISPEVEN), Mérida, Venezuela

In this cross-sectional survey, the authors assessed the relationship between metabolic syndrome and tobacco habits and found a significant association with some MS components. Here are some comments oriented to improve the manuscript:

 Introduction:

1. Paragraph 2: “ *these percentages are important because”* can be erased.

Methods:

1. Clinical evaluation: the classification of the JNC7 was not used in the results, this explanation about the JNC7 criteria can be avoided.

Results:

1. No comments.

Discussion:

1. The first paragraph should resume the main findings.
2. Paragraph 3: *“on the other hand” *can be erased.
  Paragraph 5: change “ *according to the results of our study*” for “ *In this study*”.
3. The authors should be careful in presenting cigarette use as a “protective” strategy for high blood pressure.
4. Line 13, paragraph 7: the authors should instead discuss the masked hypertension effect observed in smokers - this could explain this “paradoxical result”. This effect was also reported in patients with type 2 diabetes and smokeless tobacco use in Venezuela (González-Rivas *et al.*, 2017 ^1^).

I have read this submission. I believe that I have an appropriate level of expertise to confirm that it is of an acceptable scientific standard.

## References

[1] González-RivasJSantiagoRMechanickJNieto-MartínezR: Chimó, a Smokeless Tobacco Preparation, is Associated With a Lower Frequency of Hypertension in Subjects with Type 2 Diabetes. *International Journal of Cardiovascular Sciences*.2017; 10.5935/2359-4802.20170066 10.5935/2359-4802.20170066

# Peer review: Referee response for version 1

**DOI:** 10.5256/f1000research.15858.r33887

**Published:** 2018-05-15

**Authors:** Eiji Oda

**Affiliations:** 1 Medical Check-up Center, Tachikawa General Hospital, Nagaoka, Japan

The authors investigated cross-sectional associations between smoking and metabolic syndrome (MS) and its components in 2,212 adults from Maracaibo city, Venezuela. They reported that, in the multivariate analysis, the presence of MS, abdominal obesity, hypertriglyceridemia and hypo-HDL cholesterolemia were positively associated with current smoking while high blood pressure was inversely associated with current smoking. There was no significant association between smoking and hyperglycemia. Their data and study methods are fair. However, these results are not novel but well known findings.

 The authors stated that cigarette smoking represents an independent risk factor for the development of MS in Conclusions. However, this conclusion is incorrect because the authors stated that, regarding the limitations of this study, its cross-sectional design makes it incapable of determining causality. After all, the authors had no data regarding the association between baseline smoking status and incidence (development) of MS which can only be obtained from longitudinal studies.

I have read this submission. I believe that I have an appropriate level of expertise to confirm that it is of an acceptable scientific standard, however I have significant reservations, as outlined above.
